# Acoustic Telemetry Reveals Large-Scale Migration Patterns of Walleye in Lake Huron

**DOI:** 10.1371/journal.pone.0114833

**Published:** 2014-12-15

**Authors:** Todd A. Hayden, Christopher M. Holbrook, David G. Fielder, Christopher S. Vandergoot, Roger A. Bergstedt, John M. Dettmers, Charles C. Krueger, Steven J. Cooke

**Affiliations:** 1 Great Lakes Fishery Commission, 2100 Commonwealth Blvd. Ste. 100, Ann Arbor, Michigan, United States of America; 2 Fish Ecology and Conservation Physiology Laboratory, Department of Biology, Carleton University, 1125 Colonel By Drive, Ottawa, Ontario K1S 5B6, Canada; 3 U.S. Geological Survey, Hammond Bay Biological Station, 11188 Ray Road, Millersburg, Michigan 49759, United States of America; 4 Michigan Department of Natural Resources, 160 East Fletcher St., Alpena, Michigan 49707, United States of America; 5 Ohio Department of Natural Resources, Sandusky Fish Research Unit, 305 E. Shoreline Drive, Sandusky, Ohio 44870, United States of America; 6 Michigan State University, Center for Systems Integration and Sustainability, 1405 South Harrison Road, 115 Manly Miles Building, East Lansing, Michigan 48823-5243, United States of America; University of Girona, Spain

## Abstract

Fish migration in large freshwater lacustrine systems such as the Laurentian Great Lakes is not well understood. The walleye (*Sander vitreus*) is an economically and ecologically important native fish species throughout the Great Lakes. In Lake Huron walleye has recently undergone a population expansion as a result of recovery of the primary stock, stemming from changing food web dynamics. During 2011 and 2012, we used acoustic telemetry to document the timing and spatial scale of walleye migration in Lake Huron and Saginaw Bay. Spawning walleye (*n* = 199) collected from a tributary of Saginaw Bay were implanted with acoustic tags and their migrations were documented using acoustic receivers (*n* = 140) deployed throughout U.S. nearshore waters of Lake Huron. Three migration pathways were described using multistate mark-recapture models. Models were evaluated using the Akaike Information Criterion. Fish sex did not influence migratory behavior but did affect migration rate and walleye were detected on all acoustic receiver lines. Most (95%) tagged fish migrated downstream from the riverine tagging and release location to Saginaw Bay, and 37% of these fish emigrated from Saginaw Bay into Lake Huron. Remarkably, 8% of walleye that emigrated from Saginaw Bay were detected at the acoustic receiver line located farthest from the release location more than 350 km away. Most (64%) walleye returned to the Saginaw River in 2012, presumably for spawning. Our findings reveal that fish from this stock use virtually the entirety of U.S. nearshore waters of Lake Huron.

## Introduction

Migration is a common phenomenon in terrestrial and aquatic organisms and involves directed movements among habitats that are comparatively large relative to movements within the home range of the organism [1], [2]. In aquatic systems, migration is an important component of the life history of many fish species. Migratory behavior is often linked to obtaining transitory resources such as food, shelter, or mates [1], [2], [3]. Fish migrations include both diadromous (between marine and freshwater) and potadromous (entirely in freshwater) modalities across a broad range of spatial and temporal scales. Despite freshwater ecosystems being among the most threatened and intensively managed systems, potadromous migrations of fish in these systems are relatively unstudied [4].

The Laurentian Great Lakes of North America represent approximately 18% of the global freshwater surface supply [5]. The lakes support vibrant fish populations that generate substantial ecosystem services [6]. Despite the prominence of the Laurentian Great Lakes in North America and the ecologically and economically important fish populations they support, fish migrations in the Great Lakes have received relatively little attention. In a recent review, 88 published articles between 1952 and 2010 were identified that quantified fish movements in the Great Lakes [7]. Most of those studies employed physical marks or tags to quantify mortality rates, often with movement as a secondary question. Most of these studies were focused on a single fish species within a single lake using mark-recapture techniques, although a few recent studies have employed natural tags (e.g., otolith microchemistry) or a combination of approaches [7], [8]. Traditional mark-recapture approaches can fail to identify the full extent of migrations because of spatial limits on recapture effort such as recreational or commercial fishing effort [9], [10]. The advent of electronic fish tracking tools now enables researchers to study long-term movements of wild fish over large distances [10], [11]. Given that the Great Lakes Basin encompass multiple political boundaries and many fisheries are managed by a combination of state, U. S. tribal, or provincial agencies, knowledge of the migratory behavior and spatial ecology of fish populations that cross jurisdictional lines could improve effectiveness of fisheries management. Walleye (*Sander vitreus*) is a popular sportfish and commercially valuable fish species common to freshwater systems throughout much of the eastern United States and Canada. Known migratory components of walleye life history in the Great Lakes include seasonal migrations to shallow rocky habitats, such as offshore reef complexes or rivers and post-spawning migrations to summer feeding habitats. Historically, walleye was an apex predator that inhabited near-shore waters in Lake Huron and Saginaw Bay [12], [13]. By the mid1900s, walleye populations in Lake Huron and Saginaw Bay declined drastically because of overfishing, predation, habitat degradation, and food-web changes resulting from establishment of invasive species [14]. Following collapse of walleye populations in Lake Huron and Saginaw Bay, the fish community became dominated by invasive rainbow smelt (*Osmerus mordax*) and alewife (*Alosa pseudoharengus*). Beginning in the 1960s, Pacific salmonids were extensively stocked to control alewife and to provide recreational fishing opportunities [15], [16], [17]. Between 1999 and 2004, alewife populations declined as predatory demand by salmonids increased [12] owing to improved natural recruitment [18] and from bottom-up effects from lower food web changes resulting in decreased zooplankton abundance. As the pelagic planktivore niche declined, so did abundance of planktivores. When abundant, alewives can limit reproductive success of other species including walleye [19], [20]. Concomitant with declines in alewife abundance, the Lake Huron walleye population increased by roughly 300%, thereby meeting recovery targets and leading to cessation of stocking in 2006 [21]. Walleye emigrate from Saginaw Bay [22] and were thought to be the largest source stock contributing to local fisheries in Lake Huron and possibly western Lake Erie [23]. With recovery of the Saginaw Bay population, implications for lakewide management of the food web in Lake Huron were greater than any time since walleye collapsed in the mid-1940s.

Our objectives were to determine if migratory behavior differed between male and female walleye and to describe the timing of walleye arrival and departure from key locations (e.g., spawning river, Saginaw Bay). To achieve our objectives, we characterized spatial and temporal patterns in migratory behavior of male and female walleyes from Saginaw Bay by describing migratory pathways and the proportion of fish that migrated among multiple acoustic telemetry receiver lines in U.S. waters of Lake Huron. Walleye were tracked during one year (from April 2011 to April 2012), to enable us to characterize migration to and from spawning habitats. Knowledge of walleye migrations would increase understanding about how potadromous fish use large lakes and tributaries over large spatial (entire lake) and temporal scales. We expected male walleye would return to the spawning river before female walleye and that male walleye would inhabit the spawning river during the spawning period for a longer period of time than female walleye.

## Materials and Methods

**Figure 1:**
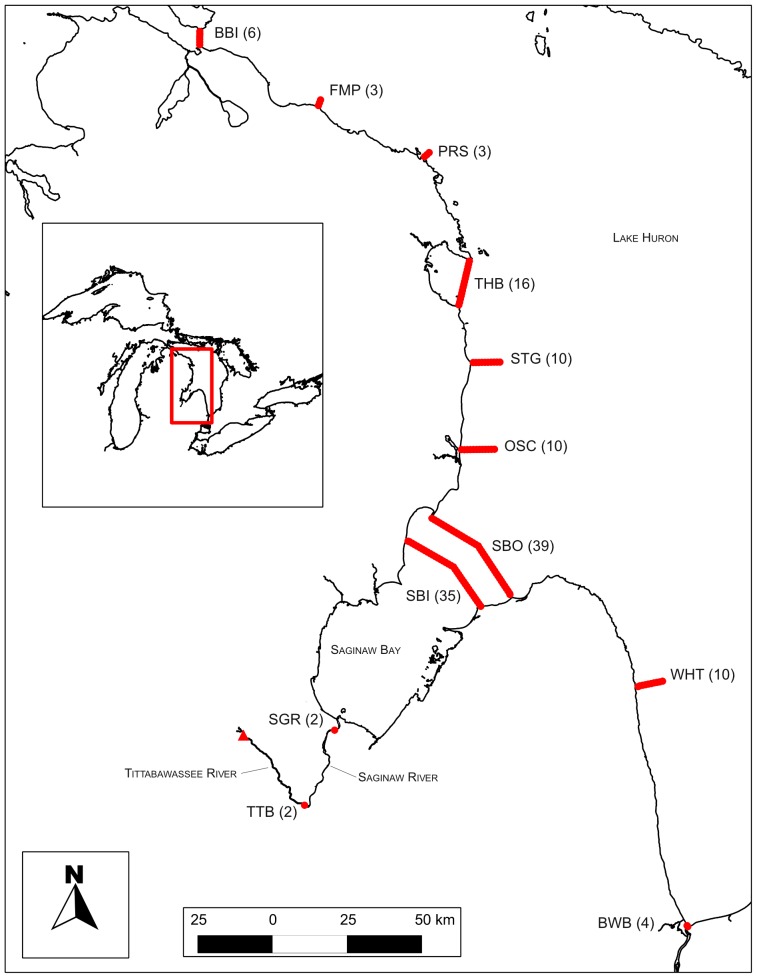
Map of study area and locations of acoustic receiver lines in Lake Huron and Saginaw Bay in 2011–2012. Inset highlights study region within the Great Lakes region. Values in parentheses denote number of receivers at each location. Walleye were tagged and released in the Tittabawassee River (triangle) in April 2011. Acoustic receiver lines: BBI – Bois Blanc Island, FMP – Forty Mile Point, PRS – Presque Isle, THB – Thunder Bay, STG – Sturgeon Point, OSC – Oscoda, SBO – Saginaw Bay outer, SBI – Saginaw Bay inner, SGR – Saginaw River, TTB – Tittabawassee River, WHT – White Rock, BWB – Blue Water Bridge.

Adult walleye were collected from the Tittabawassee River below Dow Dam (Midland, MI) using boat-mounted electrofishing gear on April 4–5, 2011 (Fig. 1). All fish were processed and tagged streamside, adjacent to the collection location. Fish selected for tagging were in spawning or post-spawning condition and were transferred to 380 L aerated holding tanks after biological data (total length, sex, dorsal spine clips) and two external t-bar anchor tags (Floy Manufacturing, Seattle, Washington) were inserted between the dorsal pterygiophores. External tags signified the presence of an internal acoustic transmitter in the event an implanted fish was harvested and enabled future accounting for fisheries mortality. Dorsal spine clips were used to estimate fish age by enumerating annual growth increments. We implanted 199 walleye, including 98 males and 101 females (mean age = 8 y, min = 2 y, max = 18 y).

Following collection of biological information and tagging, each walleye was anesthetized using a portable electroanesthesia system (PES; Smith-Root, Vancouver, Washington) operating at 35 V pulsed-direct current. A 3-second treatment induced stage-4 anesthesia for several minutes, sufficient for intracoelomic surgical implantation of the acoustic transmitter [24]. Anesthetized fish were placed in a v-shaped trough lined with soft, non-slip material and gills were irrigated with water during surgery. Surgical tools and transmitters were cleaned with povidone iodine and rinsed thoroughly with deionized water prior to surgery. Acoustic transmitters (Model V16-4H, Vemco, Halifax, Nova Scotia) were inserted through a small ventral incision located along the midline of the fish, posterior to the pelvic girdle [24]. Incisions were closed with 2–3 absorbable monofilament sutures (PDS-II, 3-0, Ethicon, Somerville, NJ). A single surgeon conducted all surgeries to reduce variation in fish survival and recovery from variations in surgical techniques [25]. Mean surgery duration was 142 seconds (min = 101 seconds, max = 212 seconds). Following surgery, fish were allowed to recover in aerated tanks and returned to the Tittabawassee River near the collection location. Postoperative recovery periods averaged ∼30 minutes and all surgical procedures followed guidelines described by Cooke et al. [26]. Acoustic transmitters (16 mm dia. ×68 mm, 24 g mass in air, est. battery life  = 1338 d) used in this study were configured to emit a tag-specific code (69 kHz) at random intervals of 60–180 s (mean  = 120 s) to reduce code collisions.

The acoustic receiver network consisted of 140 stationary receivers (69 kHz VR2W; Vemco) with omnidirectional hydrophones, deployed throughout Lake Huron during summer 2010. All receivers were retrieved and redeployed at the same location following maintenance and data download during summer 2011. Receivers were positioned in lines perpendicular to the shoreline, across bay mouths, and in rivers to monitor walleye movement in nearshore U.S. waters of Lake Huron (Fig. 1). Placement of acoustic receiver lines was based on the conceptual framework of walleye migration in Lake Huron developed from observations of jaw-tagged walleye [19]. Double receiver lines stretching across Saginaw Bay were deployed to obtain information regarding walleye movement in and out of Saginaw Bay and multiple receiver lines were used to evaluate the extent of walleye migrations in Lake Huron to biologically important habitats.

Physical characteristics of Lake Huron also influenced placement of acoustic receiver lines. Receiver lines positioned perpendicular to the Lake Huron shoreline were limited to waters less than 40 m deep and extended offshore from 3 km to 10 km. This configuration may have allowed fish to pass receiver lines while out of detection range. In water depths >2 m, an anchor-buoy system consisting of a concrete anchor connected to a buoy by stainless steel cable was used to suspend receivers 1–3 m above the lake bottom. Receivers deployed in shallow locations (<2 m) were attached to existing structure or steel anchor posts buried in the substrate. All receivers deployed in depths <3 m (one or two from each line) were removed in autumn and redeployed in spring to prevent ice-related loss or damage during winter.

Before implantation of acoustic tags, optimal receiver spacing was determined from a 10-d static-range test in Lake Huron near East Tawas, MI. A line of seven receivers spaced about 100 m apart was deployed in depths ranging 5–11 m. Two acoustic transmitters (V16-4H, Vemco) were deployed in line with receivers at depths of 2.7 m and 11.0 m to allow detection range to be calculated for 14 tag-receiver combinations representing transmitter-receiver distances from 227 to 889 m. Based on detection data, a logistic curve was used to conservatively represent the probability of detecting a single transmitter transmission as a function of range. Transmission detection probability was used in a simulation model, as described by Pincock [27], to estimate probability of detecting an implanted walleye passing through 20 simulated arrays with receiver spacing ranging 200–2000 m. In each simulation, 10,000 implanted virtual walleye swam through the simulated array at 0.25, 0.50, and 1.00 m·s^−1^ swimming speeds with random transmitter transmission intervals ranging from 120 to 360 s. Swimming speeds used in the simulation model were similar to the range of swimming speeds estimated for wild walleye [28]. The probability of detecting a fish for each array configuration was calculated as the proportion of simulated fish with two or more detections within the array. Receiver spacing of 1000 m resulted in 100% detection probability of implanted walleye using simulated data, so 1000 m receiver spacing was used for all receiver lines. All detection data for implanted fish downloaded from acoustic receivers were linked to biological data collected during the fish implanting procedure.

### Data analysis

Detection timestamps were used to estimate departure and arrival times of walleye at a receiver line. Arrival and departure times were defined as the time of first and last detection of an individual on a receiver line. The spatio-temporal pattern of detections across multiple receiver lines was used to identify direction of migration. Influence of the sex of walleye on timing and duration of migration was tested using analysis of variance. All fish detection data were pooled for analysis within a receiver line and double receiver lines located near the mouth of Saginaw Bay (SBO, SBI) were pooled for analysis (Fig. 1).

A spatial multi-state mark-recapture model [29], [30], [31] was used to quantify movement of implanted walleye among receiver lines while accounting for the possibility that implanted fish could have passed receiver lines undetected. Model structure consisted of two main pathways that were identified during initial data inspections that consisted of in-migration and out-migration components. Out-migration consisted of movements away from the release location and in-migration consisted of walleye migrations to the Saginaw River receiver line. Both pathways included out-migration from the release site to Saginaw Bay in 2011 and subsequent in-migration to the same tributaries in 2012 (Fig. 1). Pathway A reflected a northward out-migration in Lake Huron and included receiver lines located at Oscoda, Sturgeon Point, Thunder Bay, Presque Isle, Forty Mile Point, and Bois Blanc Island and in-migration to the Saginaw River (Fig. 1). Pathway B reflected a southern out-migration from Saginaw Bay and included the White Rock and Blue Water Bridge receiver lines and in-migration to the Saginaw River (Fig. 1). Model structure restricted the progression of movement to a logical sequence of receiver lines away from the release site during out-migration and back to the Saginaw River during in-migration.

A condensed detection history for each fish was used to describe movement within each pathway as a series of directed, discrete steps among receiver lines. Within each step, a tagged fish could have moved past another receiver line with probability 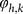 (where 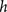
denoted previous location and 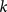
denoted next location) or ceased migration with probability 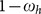, where 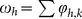 for all possible 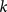. For example, from the Saginaw Bay receiver line 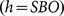, a fish could have: moved south past the White Rock receiver line with probability 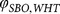; moved north past the Oscoda receiver line with probability 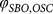; moved back into the Saginaw River with probability 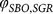; or ceased migration with probability 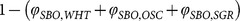. A fish could have ceased migration due to mortality or receiver avoidance (i.e., alive but remaining beyond the range of any receiver), but these causes could not be differentiated. Detection of fish beyond each line enabled estimation of site-specific detection probabilities 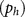, defined as the probability that a fish was detected at receiver line 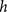 given that it passed that site. To prevent spurious results due to maximum likelihood estimation of probabilities at the boundaries of the binomial distribution (i.e., 0 and 1), we fixed detection probabilities to one in the model when the data suggested that no tagged fish passed site undetected. Detection probabilities were not estimable for the last receiver line in each migration pathway, so a “recovery” rate, 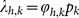, was defined as the joint probability of movement between other sites 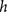
and 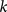 and detection at 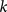.

Program MARK [32] was used to estimate parameters of the multi-state model described by Hestbeck et al. [29] and Brownie et al. [30]. The pre-defined model did not provide estimates of movement probabilities directly, but provided estimates of separate “survival” and “transition” probabilities within each step. Movement probabilities were derived as the product of “survival” and “transition” probabilities within each step. Individual survival and transition parameters were difficult to define biologically because of differences between the spatial structure of our study system and the time-based context for which multi-state mark-recapture models were originally developed. Therefore, we did not report those parameter estimates (although survival probabilities are mathematically equivalent to 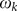 described above) and viewed them as nuisance parameters needed to estimate movement probabilities. The R package RMark [33], [34] was used to construct models for MARK. The delta method [35] was used to estimate the standard error for all derived parameters, including movement probabilities, using the R package msm [36].

Assumptions of multi-state mark-recapture models are described by Burnham et al. [37] and Skalski [38]. Model fit was assessed by estimating the overdispersion parameter 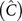 for the full model using bootstrap and median 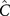 goodness-of-fit procedures in MARK [39]. The bootstrap test estimated 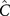 by dividing the observed full model deviance by model deviances calculated from 100 simulated datasets and the median 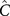 approach used logistic regression to estimate 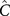. Good model fit was indicated by 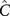 values close to 1, and as a result, Corrected Akaike's Information Criterion (AICc) values and variances were not adjusted [40].

To estimate the importance of sex on migrations of walleye at the population level, we used AICc model selection to select from multiple candidate models and to estimate migration pathway probabilities. Migration pathway probabilities 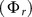 were defined as the proportion of the spawning population that used each pathway and were estimated as the product of all movement probabilities tracing each route from release (see Perry et al. 2010). For example, the proportion of the population that migrated north after leaving Saginaw Bay was calculated as: 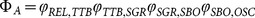. To determine if migratory behavior was a function of sex, we estimated parameters for four candidate models (Table 1). Sex was treated as a group covariate for each parameter and the most parsimonious of the candidate models was identified using AICc.

**Table 1:** Model selection results (AICc, 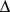
AICc, model weight, and model likelihood) for candidate models representing competing hypotheses about the effect of sex on movement 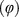 and detection probabilities (p) for walleye in Lake Huron.

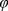	p	AICc	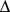 AICc	Weight	Model likelihood
–	–	1129.275	0.000	0.877	1.000
–	sex	1133.244	3.969	0.121	0.137
sex	–	1141.684	12.406	0.002	0.002
sex	sex	1146.008	16.732	0.000	0.000

Walleye were tagged and released in April, 2011 and monitored for the period of one year.

## Results

The length distribution of fish selected for tagging approximated the length distribution of the adult walleye population in Saginaw Bay. Length of tagged walleye ranged from 451 to 614 mm (mean  = 522 mm) for males and 476 to 742 mm (mean  = 557 mm) for females. Median age of tagged fish was 7 years for males and 8 years for females (min  = 4 years, max  = 18 years).

**Figure 2:**
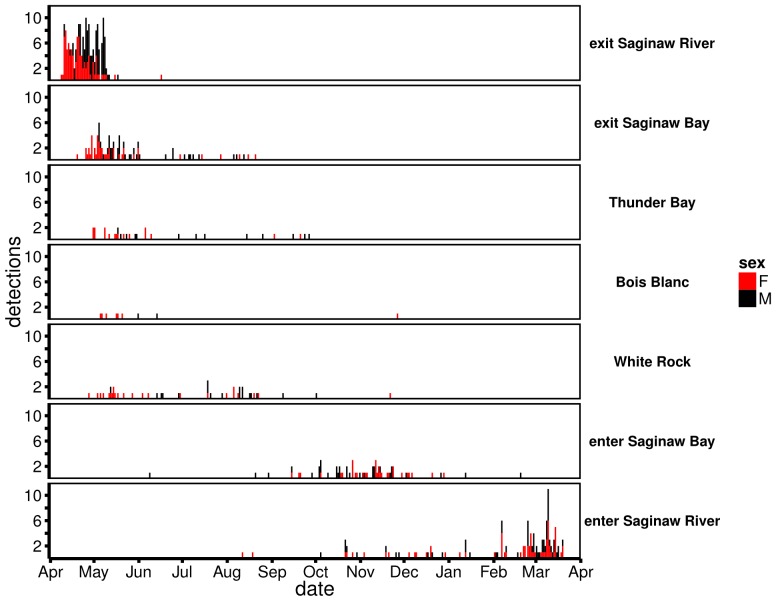
Histogram of the frequency and timing of walleye at Saginaw River, Saginaw Bay, Thunder Bay, Boise Blanc, and White Rock acoustic receiver lines (April 2011–April 2012; See **Fig. 1** for receiver line locations). Bars represent the number of walleye initially detected on a date for male (black) and female (red) walleye.

Out-migrating walleye spent an average of 21 (SE = 0.8) days in the Tittabawassee and Saginaw rivers before entering Saginaw Bay (Fig. 2). The average time spent in the Tittabawassee and Saginaw rivers differed between sexes (one-way ANOVA, *F* = 36.22, *df* = (1,187), *P*<0.0001). Male walleye spent an average of 25 days in the Tittabawassee and Saginaw rivers, whereas females spent an average of 16 days in the rivers (Fig. 2). Although walleye out-migrated from the rivers to Saginaw Bay over a 69-day period starting on April 8, 2011, more than 90% of walleye took less than 30 days to move into the bay (Fig. 2). Female walleye exited the Saginaw River and entered Saginaw Bay 9 days earlier than male walleye (ANOVA, *F* = 38.31, *df* = (1,187), *P*<0.0001).

Of fish that migrated out of Saginaw Bay and were subsequently detected on receiver lines in Lake Huron, more than 50% left Saginaw Bay by the end of May (mean departure date: May 28, 2011, SE = 3.8 days) and 90% of detected fish migrated out of Saginaw Bay by July 13, 2011 (Fig. 2). On average, female walleye exited Saginaw Bay 17 days earlier than male walleye (ANOVA, *F* = 4.904, *df* = (1, 80), *P* = 0.0296) (Fig. 2).

Walleye were detected on all Lake Huron receiver lines during the study period (April 1, 2011–April 1, 2012, Fig. 2). Although the number of walleye detected in Lake Huron varied among receiver lines, timing of detections was similar. Walleye were first detected between May 1, 2011 and December 31, 2011 on Lake Huron receiver lines (Fig. 2). The timing of first detection differed between males and females on the White Rock receiver line (ANOVA, *F* = 7.655, *df* = (1,40), *P* = 0.009) but not on the Thunder Bay receiver line (ANOVA, F = 2.85, *df* = 1, *P* = 0.102) (Fig. 2). On average, males arrived at the White Rock receiver line 38 days later than female walleye (males  =  July 26, 2011, females  =  June 18, 2011, Fig. 2).

The Bois Blanc Island receiver line, located near the Straits of Mackinac, detected nine walleye (7 female, 2 male) during the study (Fig. 2). All but one walleye were detected at the Bois Blanc Island receiver line over a three-month period starting on May 5, 2011 (Fig. 2). The first walleye detected on the Bois Blanc Island receiver line moved more than 350 km in 30 days. On average walleye moved from release to the Bois Blanc Island receiver line in 49 days and moved from Bois Blanc Island to the Saginaw River in 170 days.

In-migrating walleye returned to Saginaw Bay over a seven-month period starting in August 2011. The daily return rate to Saginaw Bay was less than 2 fish per day before October 1, 2011 and peaked during a 35-d period starting on October 16, 2011 (Fig. 2). Over 90% of in-migrating walleye entered Saginaw Bay by December 5, 2011 (Fig. 2). The timing of in-migration to Saginaw Bay did not vary among pathways (i.e., northern or southern) or between sexes (two-factor ANOVA, *df*_sex_ *= *(1,62), *F*_sex_ *= *2.740, *P*_sex_ = 0.103, *df*_path_ *= *(1,62), *F*_path_ *= *2.138, *P*_path_ = 0.149, *df*_sex*path_ *= *(1,62), *F*_sex*path_ *= *0.002, *P*_sex*path_ = 0.968).

Walleye were detected entering the Saginaw River between August 1, 2011 and April 1, 2012, but more than 50% of fish were first detected between January 14, 2011 and March 9, 2012 (Fig. 2). The daily return rate to the Saginaw River was less than 2 individuals before February 2012, and thereafter, peaked between February and April 2012Walleye that did not leave Saginaw Bay and in-migrated to the Saginaw River returned 28 days earlier than walleye that followed the southern Lake Huron migratory pathway and 35 days earlier than walleye that followed the northern Lake Huron migratory pathway (two-factor ANOVA with Tukey's HSD Post Hoc test, *F*_sex_ = 0.002, *df*_sex_ = (1,113), *P*_sex_ = 0.961, *F*_path_ = 6.361, *df*_path_ = (2,113), *P*_path_ = 0.002, *F*_sex*path_ = 1.487, *df*_sex*path_ = (2,113), *P*_sex*path_ = 0.230) (Figs. 1,2). Timing of Saginaw River entry did not differ between fish that migrated along the northern or southern migratory pathways.

The most parsimonious model in the analysis of migratory pathways using multi-state mark recapture models did not include fish sex, which suggests that migratory behavior did not differ between sexes (Table 1). The smallest 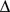
AICc value comparing all candidate models in our study was 4.0; 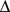
AICc values >2 and <7 represent substantial support for real differences between the models [40] (Table 1). The best model was 7.3 times more likely than the next model (Table 1). Estimates for 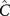 were 1.06 using the bootstrap approach and 0.91 (SE = 0.042) using the median 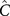 test. Deviance residuals for the most parsimonious model were randomly distributed and ranged from −2.0 to 2.0. Modeled Detection probabilities ranged from 0.80 to 1.00 among receiver lines (Table 2).

**Table 2:** Estimated detection probabilities (p) for acoustic receiver lines in Saginaw Bay and Lake Huron from April 2011 to April 2012 as a function of movement direction.

Receiver line	Migration direction	p (SE)
TTB	Out	*1*
SGR	Out	*1*
SGR	In	*1*
SBO	Out	0.988 (0.012)
OSC	Out	0.828 (0.064)
STG	Out	0.968 (0.031)
THB	Out	*1*
PRS	Out	*1*
FMP	Out	*1*
WHT	Out	*1*

Detection probabilities were estimated for the candidate model with the lowest AICc value. In-migration is the return of tagged walleye to the Saginaw River (SGR) and out-migration is migration away from the Tittabawassee River release location. Estimated detection probabilities approaching one were fixed to one in model (italicized). Acoustic receiver lines: TTB – Tittabawassee River, SGR – Saginaw River, SBO – Saginaw Bay outer, OSC – Oscoda, STG – Sturgeon Point, THB – Thunder Bay, PRS – Presque Isle, FMP – Forty Mile Point, WHT – White Rock. See Fig. 1 for locations of receiver lines.

**Figure 3:**
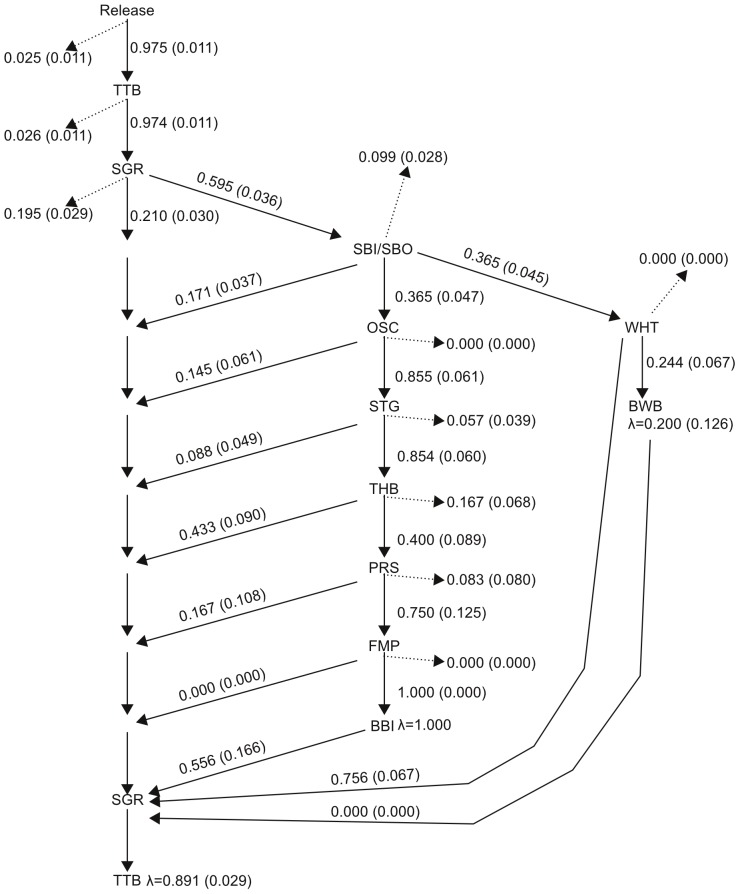
Movement probabilities for two migration pathways estimated from a multi-state mark-recapture model for tagged walleye released in the Tittabawassee River in 2011. The northern pathway consists of movements from the Saginaw Bay (SBI/SBO) receiver line to the Oscoda (OSC), Sturgeon Point (STG), Thunder Bay (THB), Presque Isle (PRS), Forty Mile Point (FMP), or Bois Blanc Island (BBI) receiver lines. The southern pathway consists of movements to White Rock (WHT) and Blue Water Bridge (BWB) receiver lines from the Saginaw Bay (SBI/SBO) receiver line. See Fig. 1 for receiver line locations. Solid arrows represent the probability of moving between receiver arrays, given that a fish survives and is present. Dashed lines represent the probability that a fish detected at the location ceased migration after detection. Values in parentheses are standard error of the estimate.

Out-migration rates from the Tittabawasse and Saginaw rivers were similar. Greater than 97% of released walleye were detected on the Tittabawassee River receiver line and 99% of fish detected on the Tittabawassee receiver line entered Saginaw Bay (Fig. 3). Overall, 95% (SE = 1.5%) of tagged walleye out-migrated to Saginaw Bay after release. About 5% of implanted walleye ceased migration along modeled migratory pathways and did not enter Saginaw Bay. Of walleye that entered Saginaw Bay, 59.5% (SE = 3.6%) were detected on the Saginaw Bay receiver line at the eastern edge of Saginaw Bay; and 21.0% (SE = 3.0%) that did not leave Saginaw Bay in-migrated to the Saginaw River after at least 60 days at large (Fig. 3). About 19.5% (SE = 2.9%) of walleye that entered Saginaw Bay were not detected on any other receivers.

Most (>50%) fish detected on the Saginaw Bay receiver line at the mouth of Saginaw Bay migrated out of Saginaw Bay and were subsequently detected on Lake Huron receiver lines. From the Saginaw Bay receiver line (SBI & SBO; Fig. 1), 36.5% (SE = 4.7%) of walleye migrated along the northern pathway and 36.5% (SE = 4.5%) migrated along the southern pathway (Fig. 3). About 27% of walleye detected on the Saginaw Bay receiver line did not follow northern or southern migratory pathways. Of these fish, 17.1% (SE = 3.7%) returned to the Saginaw River receiver line in 2012 and 9.9% (SE = 2.8%) were not detected on any other receivers (Fig. 3). From release, less than 10% of fish migrated out of Saginaw Bay and were not detected on any receiver lines before returning to the Saginaw River in 2012 (Fig. 3). These fish likely represent unknown or undescribed migratory pathways.

The percentage of tagged walleye that followed the northern migratory pathway decreased with increasing distance from the release location. About 20% (SE = 2.9%) of released walleye were detected on the Oscoda receiver line, 18% (SE = 2.7%) were detected on the Sturgeon Point receiver line, 15% (SE = 2.5%) were detected on the Thunder Bay receiver Line, 6% (SE = 1.7%) were detected on the Presque Isle receiver line, and 4.5% (SE = 1.5%) were detected on Forty Mile Point and Bois Blanc Island receiver line (Fig. 3). Walleye that out-migrated from the release site and were detected about 50% less frequently on receiver lines north of Thunder Bay (Presque Isle, Forty Mile Point, Bois Blanc) than on receiver lines south of Thunder Bay. Except for the Thunder Bay receiver line, the percentage of walleye detected on each receiver line that ceased movement along the northern migration pathway was less 10.0% (Fig. 3). The proportion of walleye that ceased movement along the northern migration pathway after detection on the Thunder Bay receiver line was approximately two times higher than any other receiver line (Fig. 3). The percentage of tagged walleye detected was 20.6% (SE = 2.9) on the White Rock receiver line and 5.0% (SE = 1.5%) on the Blue Water Bridge receiver line (Fig. 3). All walleye detected on the White Rock receiver line continued to move along the southern migration pathway.

Overall, 63% (SE = 3.5%) of walleye that out-migrated after release returned to the Saginaw River mouth in 2012. Individual in-migration probabilities between receiver lines ranged from 8% to 56% for the northern migratory pathway and from 0.0% to 7.5% for the southern migratory pathway (Fig. 3). With the exception of the Bois Blanc Island and Thunder Bay receiver lines, individual in-migration probabilities were less than 17% (Fig. 3). Higher in-migration probabilities at the Thunder Bay and Bois Blanc Island receiver lines than other receiver lines on the northern Lake Huron migratory pathway suggest unknown and/or undescribed migration pathways. In-migration probabilities of walleye that migrated along the southern pathway were highly variable. Approximately 76% (SE = 6.7%) of walleye detected on the White Rock receiver Line in-migrated to the Saginaw River (Fig. 3). However, no walleye (0%) that were detected on the Blue Water Bridge receiver line returned to the Saginaw River in 2012 (Fig. 3).

## Discussion

Walleye moved extensively from their tagging location in the Tittabawassee River throughout U. S. waters of Lake Huron. Most walleye also returned to their spawning river one year later. The timing of post-spawn walleye movements in the Tittabawassee and Saginaw rivers differed by sex in our study. Female walleye spent less time in the Tittabawassee and Saginaw rivers while migrating to Saginaw Bay and were detected on Lake Huron receiver lines earlier than male walleye. Our results are consistent with the observations by Madenjian et al. [41] who documented higher contaminant levels in male Saginaw Bay walleye and attributed that to longer river residence time as compared to females. Male walleye may maximize reproductive success by repeat spawning with multiple females such that spending more time at the spawning grounds results in additional spawning opportunities. The higher energetic costs of egg production for female walleye may limit time spent on the spawning grounds and, therefore, the optimal behavioral strategy for females may trade off time on spawning grounds with prey acquisition in Saginaw Bay or Lake Huron.

Our estimate of the proportion of post-spawn walleye that immigrated to Lake Huron and the influence of sex on walleye migration contradict results from a multi-year tag recovery study in Lake Huron. Estimates of immigration to Lake Huron for jaw-tagged walleye captured and released at the same location as our study averaged 8.8% between 1981 — 2011 compared to 56.5% of walleye detected at the mouth Saginaw Bay in our study [42]. A greater proportion of jaw-tagged female walleye were recaptured in Lake Huron than male walleye, in contrast to our study where sex did not influence walleye movement to the lake [43]. In Lake Erie, movement of jaw-tagged walleye recaptured by commercial fishing suggested that sex influenced walleye movement patterns and movements were linked to water temperature and obtaining prey resources [44], [45], [46]. Saginaw Bay water temperatures often exceed the thermal preferences of walleye and may be the motivation for immigration to Lake Huron despite higher prey density in Saginaw Bay compared to Lake Huron [47], [21].

About 64% of tagged and released walleye returned to the Saginaw River in 2012, presumably for spawning. Spawning site fidelity of walleye in Lake Huron has been documented; estimates of the proportion of the population that exhibit this behavior are not well known [48], [49], [50], [51]. The moderate level of spawning site fidelity observed in our study is consistent with evidence from genetic investigations that support a single panmictic walleye population in Lake Huron [52]. Our estimates of the proportion of individuals that returned to the Saginaw River were similar for all detection locations, regardless of the location or distance between the location where a fish was detected and the Saginaw River mouth, indicating that return to the Saginaw River is a directed movement. We could not determine the fate of individuals that did not return to the Saginaw River in 2012, although natural or fishery-related mortality, selection of alternative spawning location, transmitter failure, or a reproductive holiday are plausible explanations that will require further investigation [53]. Our results are consistent with the conceptual model of spawning migrations, characterized by postspawn movements to Saginaw Bay and Lake Huron and return of walleye to the same spawning location across multiple years [54], [55], [56]

High (>0.80) detection probabilities for all monitoring lines suggest walleye inhabited waters that were monitored with acoustic receivers in Lake Huron and receiver lines sufficiently sampled acoustic transmissions. Detection probabilities represent the ratio of fish detected at a receiver line to those detected at all subsequent receiver lines, assuming fish encounter receivers in succession [57]. Detection of a fish at a receiver line depends on many factors, including environmental conditions, spatial configuration of the acoustic transmitter and receiver pair, number of receivers at a location, and behavior of the fish [58]. In our case, a walleye tagged in the Tittabawassee River must sequentially pass Tittabawassee River, Saginaw River, and Saginaw Bay monitoring lines before reaching Lake Huron. In contrast, acoustic monitoring lines in Lake Huron were configured as a line of nearshore receivers situated perpendicular to the shoreline, permitting fish to potentially bypass receiver lines in depths >40 m. Given this arrangement, Lake Huron detection probabilities reflect undetected fish that could have been detected at a monitoring line or fish that avoided the nearshore monitoring line. Given that fish can bypass receiver lines after exiting Saginaw Bay, our estimate of the proportion of walleye that migrated out of Saginaw Bay using our multi-state model was likely conservative. Indeed, 10% of the fish tagged migrated to outer Saginaw Bay and were not encountered anywhere else before returning to the Saginaw River. We could not determine if these fish migrated out of Saginaw Bay or avoided detection within the bay receiver lines, although it is likely that some of these fish migrated out of the bay.

### Implications for fishery management

Migratory behavior of walleye has important implications for fishery management. The Saginaw Bay walleye fishery does not operate on a closed population and fishing success in northern or southern regions of Lake Huron are linked to fish spawning in Saginaw Bay as evidenced by Saginaw Bay jaw tags being reported by the recreational fishery outside the bay [42] and confirmed by our telemetry findings. If walleye migrations out of Saginaw Bay is linked to walleye density in the bay, then increasing walleye population density in the bay may strengthen links to northern and southern Lake Huron. Understanding links between Saginaw Bay and Lake Huron is important for development of stock-specific management practices, such as manipulating harvest with specific populations. Greater proportions of Saginaw Bay walleyes are emigrating from the bay than indicated by jaw tag returns and thus greater proportions of Lake Huron's walleye fisheries are likely comprised of Saginaw Bay walleyes. Successful management of this stock of walleyes will require understanding the collective effect of these fisheries around the lake.

We evaluated whole-lake migration of a freshwater piscivore and did so in the third largest freshwater lake by surface area in the world. Studies of this scale have only recently been possible in deep freshwater lakes because of developments in automated acoustic telemetry systems [11]. Not only did we show that adult walleye migrate along distinct pathways in Lake Huron, we generated some of the first information on potadromous fish behavior for a species that is subject to fishery exploitation. Our analysis, however, was limited to a single year of observations, so migratory behavior we found may not be repeatable from year to year. Future work is needed to determine consistency of patterns across years, particularly for individual fish. In addition, expansion of the telemetry network into eastern Lake Huron, placement of acoustic receivers at other spawning tributaries, and tagging of fish from other tributaries would further elucidate meta-population dynamics of walleye at a grand scale. Even more broadly, understanding migration of walleye among Great Lakes, such as between Lake Huron and Lake Erie, will provide a clearer picture of the scope and scale at which walleye populations mingle in the Great Lakes.

## Supporting Information

S1 Table **Date and time of arrival and departure of each tagged walleye at receiver lines in Saginaw Bay and Lake Huron in 2011–2012.** Walleye were tagged and released in the Tittabawassee River in April 2011. Sex (F – female, M – male) was determined for all tagged walleye and each fish was assigned a unique identification number (id). Arrival (arrive) and departure (depart) timestamps represent the first and last detection of each walleye on each receiver lines. Timestamps are reported in year-month-day hour-minute-second format in coordinated universal time (UTC) timezone. See Fig. 1 for receiver line locations. Acoustic receiver lines: BBI – Bois Blanc Island, FMP – Forty Mile Point, PRS – Presque Isle, THB – Thunder Bay, STG – Sturgeon Point, OSC – Oscoda, SBO – Saginaw Bay outer, SBI – Saginaw Bay inner, SGR – Saginaw River, TTB – Tittabawassee River, WHT – White Rock, BWB – Blue Water Bridge.
(CSV)

S2 Table **Frequency of individual detection histories for male and female walleye in Lake Huron and Saginaw Bay.** Detection history represents walleye movements along migratory pathways consisting of 12 detection occasions. See Fig. 3 for model schematic, detection locations, and detection occassions. Values of “A”, “B”, “C” in individual detection history represent walleye detection and movement along migration pathway. Values of 0 in detection history represent no detection during the occasion. Frequency is the number of walleye that exhibited each detection history.
(CSV)
